# Novel *ITGB2* Mutation Is Responsible for a Severe Form of Leucocyte Adhesion Deficiency Type 1

**DOI:** 10.1155/2022/1141280

**Published:** 2022-03-03

**Authors:** Ahmed Bouhouche, Yasmin Tabache, Omar Askander, Hicham Charoute, Nada Mesnaoui, Lamiae Belayachi, Naima El Hafidi, Houyam Hardizi, Elmostafa El Fahime, Naima Erreimi, Abdelhamid Barakat, Mohammed Khattab, Fouad Seghrouchni, Amine El Hassani

**Affiliations:** ^1^Research Team in Neurology and Neurogenetics, Genomics Center of Human Pathologies, Medical School and Pharmacy, Mohammed V University in Rabat, Morocco; ^2^Research Genetics Center of the Cheikh Zaid Foundation, Abulcasis International University of Health Sciences, Rabat, Morocco; ^3^Department of Pediatrics, Abulcasis International University of Health Sciences, Rabat, Morocco; ^4^Centre of childhood Care and Prevention, Cheikh Zaid International Universitary Hospital, Rabat, Morocco; ^5^Research Unit of Epidemiology, Biostatistics and Bioinformatics, Institut Pasteur du Maroc, Casablanca, Morocco; ^6^Department of Pediatric Children Hospital, Medical School and Pharmacy, Mohammed V University in Rabat, Morocco; ^7^Molecular Biology and Functional Genomics Platform, National Center for Scientific and Technical Research, Rabat, Morocco; ^8^Laboratory of Genomics and Human Genetics, Institut Pasteur du Maroc, Casablanca, Morocco; ^9^Laboratory of the Cellular Immunology, Institut National d'Hygiène, Rabat, Morocco

## Abstract

Leukocyte adhesion deficiency type 1 (LAD1) is a rare autosomal recessive hereditary disorder characterized by recurrent infections, impaired pus formation, delayed wound healing, omphalitis, and delayed separation of the umbilical cord as hallmark features of the disease. It results from mutations in the integrin *β*2 subunit gene *ITGB2*, which encodes the integrin beta chain-2 protein CD18. In this study, we aimed to investigate the case of a five-month-old boy who presented with a clinical phenotype and flow cytometry results suggesting LAD1 disease. Sanger sequencing of all exons and intron boundaries of *ITGB2* identified a novel in-frame deletion in exon 7 (*ITGB2* c.844_846delAAC, p.Asn282del) in the patient. The p.Asn282del mutation was heterozygous in the child's parents, whereas it was absent in the 96 control individuals from North Africa. This variant was evaluated by two in silico mutation analysis tools, PROVEAN and MutationTaster, which predicted that the mutation was likely to be pathogenic. In addition, molecular modeling with the YASARA View software suggested that this novel mutation may affect the structure of integrin beta-2 and, subsequently, its interaction with integrin alpha-X. In summary, we report a novel pathogenic mutation p.Asn282del associated with LAD1 that expands the mutation diversity of *ITGB2* and suggest the combination of flow cytometry and *ITGB2* sequencing as a first-line diagnostic approach for LAD disease.

## 1. Introduction

Leukocyte adhesion deficiency (LAD) is a primary immune deficiency disorder with an autosomal recessive mode of inheritance, caused by loss of function of the differentiation cluster 18 (CD18) [1]. This defect leads to dysfunction of leukocyte adhesion to the wall of blood vessels and migration of leukocytes to sites of infection and inflammation. Patients with LAD are infected with common pathogenic agents but not opportunistic ones and should respond well to antimicrobial therapy. Proteus, Klebsiella, Staphylococcus aureus, Pseudomonas aeruginosa, and enterococci are the most common pathogens affecting LAD patients [2]. LAD includes four subtypes, LAD1, LAD2, and LAD3 and LAD4, of which LAD1 is the most frequent [3]. LAD1 is characterized by recurrent infections, impaired pus formation, delayed wound healing, omphalitis, and delayed separation of the umbilical cord, which usually occur in the neonatal period. Periodontitis, pneumonia, colitis, sepsis, cervical lymphadenopathy, and osteomyelitis are other clinical manifestations of the disease [3]. This severe disease remains a life-threatening condition with limited 2-year survival in the absence of allogeneic hematopoietic stem cell transplantation [4].

The tethering and adhesion of polymorphonuclear neutrophils and monocytes to the blood vessel's endothelium upon an acute inflammatory response require involvement of adhesion molecules. Initial tethering and rolling are mediated by the selectin molecules, whereas transmigration to the tissue is mediated by the adhesion of leucocytes with *β*2 integrins that are constitutively present on their plasma membranes [5]. Then, deficiencies in CD18 prevent normal integrin dimerization and leukocyte adhesion to endothelial surfaces, processes essential to extravasation and antimicrobial activity [6, 7].

LAD1 results from mutations in the integrin *β*2 subunit gene (*ITGB2*, OMIM: 600065), located at the long arm of chromosome 21q22.3 [8]. It contains 17 exons and encodes the integrin beta chain-2 protein, also called CD18, which forms a heterodimer with each of the alpha chains (CD11a, CD11b, CD11c, or CD11d). Defects in CD18 could affect the expression of all *β*2 integrins, leading to the clinical features of LAD1 [9].

Several different mutations have been identified in *ITGB2*, including missense, nonsense, splice, and INDEL mutations. Most of them occur in exons 5 to 9 that encode the *β*1 domain of ITGB2. *ITGB2* mutations affect the expression of *β*2 integrins on the leukocyte surface. Therefore, CD18 expression has been commonly used as a marker for clinical diagnosis and severity classification of LAD1. Patients with 1% expression were described as severe, while those with 2.5% to 10% expression were moderate to mild [10]. When the clinical phenotype suggests LAD, flow cytometry analysis of CD18 and CD11 (a, b, and c) expression on peripheral blood leukocytes is a rapid and sensitive tool commonly used for diagnostic confirmation [11].

To date, reports of LAD diagnoses worldwide are only in the hundreds, with a low incidence of 1/10 million [1]. Most of these patients originated from Asia, especially India and Iran [4]. In North Africa, only five cases originating from Tunisia have been identified [12]. In this study, LAD disease was suspected in a North African child from Mauritania, and the diagnosis was confirmed by flow cytometry. Sanger sequencing of the ITBG2 gene identified a novel homozygous mutation in exon 7, p.Asn282del. This nonframeshift deletion was predicted pathogenic by in silico tools and molecular modeling.

## 2. Materials and Methods

### 2.1. Subject Evaluation

**Figure 1:**
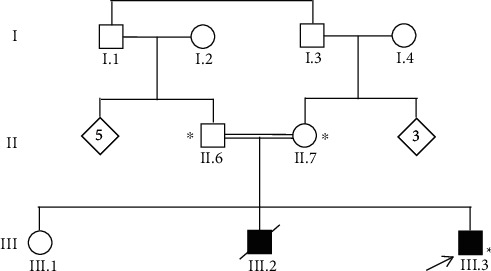
Pedigree of the consanguineous Mauritanian family studied. Arrow: index patient. Asterisk: genetic testing performed.

A five-month-old male infant from Mauritania (native and living) was admitted to the pediatric department of Cheikh Zaid Hospital for etiological assessment of recurrent infections. The family tree was built after a family survey of the parents, identifying the infant's brother died at the age of eight months with the same history of the disease and one healthy older sibling (Figure 1). Parents are healthy and first-degree consanguineous, suggesting an inherited disease with an autosomal recessive mode of inheritance.

The blood samples were collected from the patient and their parents. Signed informed consent was obtained from all subjects or their legal guardians for these studies in accordance with the Helsinki principles, and the study protocol was approved by the local ethics committee of Cheikh Zaid Hospital (ID: CEFCZ/AB/2021PR_RFG).

### 2.2. Flow Cytometry

Lymphocyte subpopulation exploration was determined in whole peripheral blood by lysing, no-wash flow cytometry protocol. The mixture of fluorochrome-labeled monoclonal antibodies is as follows: CD3, FITC, CD16 PE + CD56 PE, CD45 PerCP-Cy™5.5, CD4 PE-Cy™7, CD19 APC and CD8 APC-Cy™7 (Multitest™ 6-color TBNK, BD BioSciences). The mixture DR-HLA-FITC/CD19-PE/CD45 PerCP-Cy™5.5 (all from BD Biosciences) was used to measure the expression of DR-HLA molecules by B cells. The amount of alpha-beta double-negative T cells subpopulation was estimated using the mixture CD3-FITC/TCRalpha-betta-PE/PerCP-Cy™5.5/CD4+CD8-APC (all from BD Biosciences). The percentage of each targeted lymphocyte subpopulation was directly given by the cytometer analysis software, while the absolute sizes were determined using the True Count® beads (BD Biosciences).

The expression of integrin molecules by leucocytes was measured in peripheral whole peripheral blood by lyse-wash flow cytometry immunophenotyping protocol. This expression intensity was evaluated using monoclonal antibodies directed against CD11/CD18 complex. Thus, the whole blood sample was stained by the mixture CD11c PE/CD18 FITC (all from BD Biosciences, CA, USA). Measurement was performed to identify and estimate the percentage of positive fluorescent neutrophils and monocytes and the ratio of median fluorescence intensity (MFI) of the stained to unstained tubes.

The quantitative determination of neutrophil oxidative burst was performed using the Phagoburst™ kit (BD Biosciences) as recommended by the manufacturer. Sample acquisition was performed using a FACS Canto II cytometer (BD BioSciences), and data analysis was done using FlowJo v10.0.7 software (Tree Star, Inc.).

### 2.3. Genetic Analysis

The blood samples were taken from the patient and their parents, and high-quality genomic DNA was purified from peripheral blood leukocytes using the Wizard® Genomic DNA Purification Kit from Promega. The quality and concentrations of DNA samples were examined by Nanodrop 1000 Spectrophotometer (Thermo Fisher Scientific). We sequenced all exons and flanking intronic sequences of *ITGB2* using the primers and PCR conditions of Yassaee et al. [13]. PCR products were sequenced using BigDye™ Terminator v3.1 Ready Reaction Cycle Sequencing Kit, electrophoresis was performed on SeqStudio Genetic Analyzer, and sequence chromatograms were analyzed by SeqScape 4 software (Thermo Fisher Scientific). Ninety-six DNA samples from healthy individuals all of Moroccan origin were sequenced for *ITGB2* exon 7 to verify the frequency of the novel mutation identified.

### 2.4. Bioinformatics Analysis

The in silico mutation analysis tools, PROVEAN and MutationTaster, were applied to predict the pathogenicity of the novel mutation. Moreover, a molecular modeling analysis was performed to predict the effect of the p.N282del amino acid deletion on the integrin beta-2 protein. The tridimensional structure of the wild-type integrin beta-2 in complex with integrin alpha-X (CD11c) was retrieved from the Protein Data Bank (PDB ID: 3K6S). The 3D structure of this protein complex upon integrin beta-2 mutation was predicted using the homology modeling web server Swiss Model. Next, the energies of both complexes (native and mutated) were minimized using the Yasara Energy Minimization server. Hydrogen bonds and hydrophobic interactions were analyzed using the YASARA View software. This bioinformatics tool was also used to calculate the structural distance between native and mutated protein complexes.

## 3. Results

### 3.1. Clinical Report

The studied patient III.3 was an infant born at term following a well-attended pregnancy with a birth weight of 3400 g, displaying good adaptation to extrauterine life. He has been exclusively breastfed since birth. Umbilical cord separation was delayed, occurring at four weeks. He received the hepatitis B vaccine at birth, his BCG vaccine on day 7 of life, and two doses of vaccine against poliomyelitis, diphtheria, whooping cough, and tetanus, without the third recall in the 3rd month. There was no allergy or any exposure to toxic agents.

The symptoms started on day four of life by the successive occurrence of several episodes of infection. The first episode occurred and was resolved with symptomatic treatment of the fever only. The second one at one month necessitated hospitalization and intravenous antibiotics and was characterized by a high temperature with a positive inflammatory syndrome. The third episode occurring at two months in the form of pyodermatitis was treated with local and oral antibiotics. At the age of three months, a new infectious episode occurred with gingivostomatitis associated with bronchopulmonary infection and a positive inflammatory syndrome (hyperleukocytosis at 80,000 elements/mm3 and elevation of the C-reactive protein) also treated by intravenous antibiotics. The fifth and last episode at four months consisted of fever and a persistence of hyperleukocytosis at 80,000 elements/mm^3^ for which antibiotics were given but showed no improvement. The parents also reported chronic liquid diarrhea (four stools per day) without rectorrhagia or other associated signs.

Following admission to our structure, the clinical examination found a conscious, tonic, reactive, hemodynamically, and respiratory stable infant, without fever. His weight was 6550 g (-2 SD), his size was 65 cm (M), and his cranial perimeter was 43 cm (+1 SD). There was a cutaneous mucosal pallor, without rash, or peripheral cyanosis or hemorrhagic signs (bruising, purpura) or cutaneous ulceration. Oral thrush was found as well as hair scarcity. The rest of the examination was normal.

The blood count showed hyperleukocytosis at 44,020 elements/mm^3^ with polymorphonuclear neutrophils at 39% (i.e., 17,555 elements/mm^3^), 50% lymphocytes (i.e. 22,411 elements/mm^3^), 3.4% eosinophils (i.e. 1,497 elements/mm3), 0.13% basophils (i.e. 1,497 elements/mm^3^), and 5% monocytes (i.e. 24,483 elements/mm). Normochromic normocytic anemia was found with hemoglobin at 10.45 g/dl, and the platelet level was 516,000 elements/mm^3^. The blood smear revealed the presence of 28% activated lymphocytes with no abnormal cells.

The C-reactive protein was at 4.4 mg/l, with a negative procalcitonin (0.06 ng/ml), a fibrinogen level at 4.58 g/l, and normal ferritinemia. A coproparasitology with a search for rotavirus in the stools was negative, the cytobacteriological examination of urine was negative, and the blood serum ionogram was normal. The immunoglobulin assay showed a level of IgG at 10.6 g/l, IgM at 1.03 g/l, Ig A at 0.7 g/l, and the level of total Ig E at 122.44 IU/ml. HIV and CMV serologies were negative. Finally, a radiological check did not show any abnormalities (chest X-ray, abdominal ultrasound, and chest-abdominal CT).

The patient was born to first-degree consanguineous parents, without any pathological history. Brother III.2 died at the age of eight months from septic shock with a cutaneous starting point and had a notion of recurrent infections and delayed cord fall. The older brother III.1, aged 4 years, is healthy. There were no known hereditary pathologies in the family. Therefore, the clinical presentation in patients of the studied family suggested LAD1 disease.

### 3.2. Flow Cytometry Results

Lymphocyte subpopulation quantification within the patient III.3 showed normal percentage and absolute size of T lymphocytes (61%, 7005 cells/*μ*l), CD4 T cells (47.9%, 5054 cells/*μ*l), CD8 T cells (10.6%, 1022 cells/*μ*l), and B cells (20.5% 2037 cells/*μ*l) compared to the ranges of the respective normal values corresponding to zero to one-year-old children (T cell: 3200-7900 cells/*μ*l, CD4T 2000-6100, CD8T cells 900-2200, and B cells: 460-3630 cells/*μ*l) [14]. However, the NK cells showed a relatively elevated percentage and absolute size (17.6%, 2003 cells/*μ*l) compared to the corresponding normal range (50-1640 cells/*μ*l). On the other hand, no deficiency was observed in the expression of DR-HLA molecules, since 100% of B cells express DR-HLA molecules on their surface. No abnormality was observed in the amount of alpha-beta double-negative T cells subpopulation (0.1% of T cells).

Neutrophils also showed a normal oxidative burst activity. Almost all these cells were engaged in this activity following stimulation by PMA (100%) as well as by opsonized E. coli (98%).

**Figure 2:**
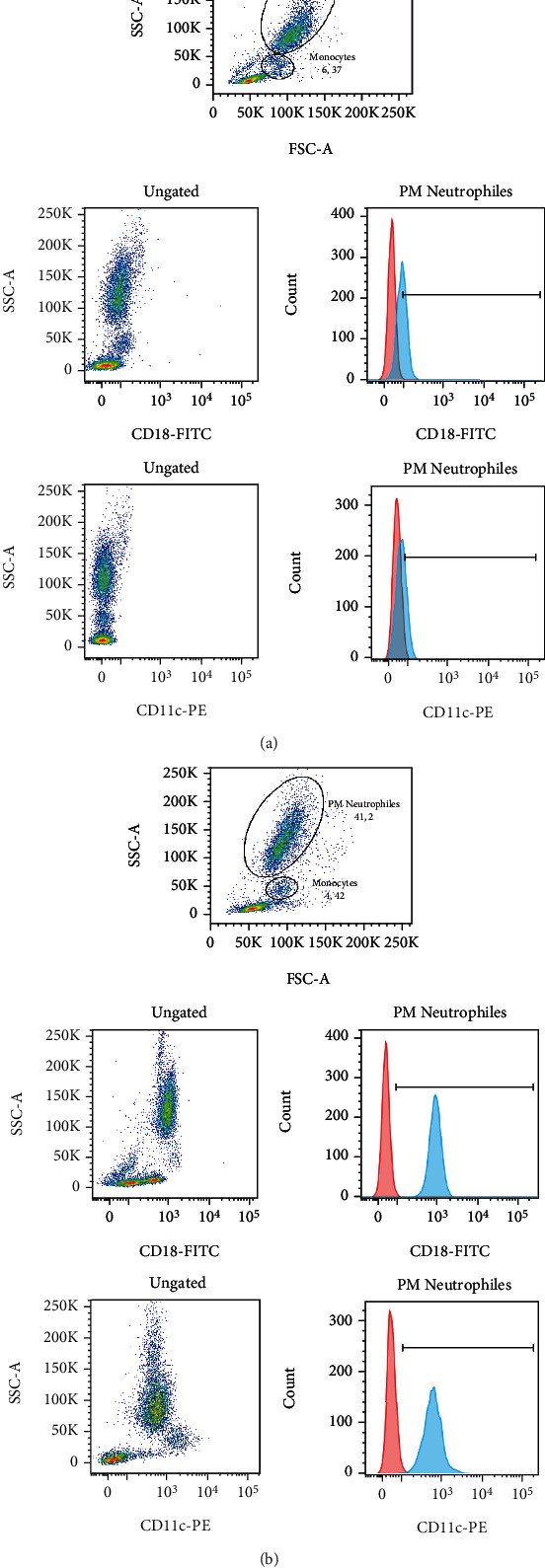
Dot plots of flow cytometry analysis of CD18 and CD11c expressions on neutrophils and monocytes within the patient with LAD1 (a) and a normal healthy control tested in the same batch (b). Histograms of neutrophil fluorescence are represented. The ratio of MFI is obtained by the ratio of the medians of the stained (blue) histograms to the unstained (red) histograms. The percentages represent the rate of positive fluorescent neutrophils.

However, the patient showed a significantly reduced expression of integrin molecules on both neutrophils and monocytes compared to the normal control tested in the same batch (Figure 2). In the surface of neutrophils, this reduction concerns the expression of both CD18 (1.51% vs 99.9% and MFI ratio: 7.7 vs 46.2) and CD11c (0.21% vs 99.6% and MFI ratio: 3.3 vs 24.6).

### 3.3. Genetic Findings

**Figure 3:**
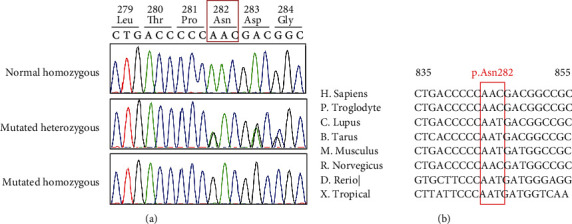
Sanger validation and phylogenetic conservation of the novel missense mutation in ITGB2. Sanger sequencing confirms the presence of the c.844_846delAAC mutation in ITGB2 in heterozygous and homozygous states (a). Partial nucleotide sequence alignment of human ITGB2 with orthologs shows evolutionary conservation between species of the codon 282 (b). The red box indicates the triplet of nucleotides deleted in the patient.

We report a case of a five-month-old male of Mauritanian origin with clinical and biological features consistent with LAD1 disease. Sanger sequencing of all the exons and flanking introns of *ITGB2* showed a homozygous INDEL mutation of three nucleotides in exon 7, c.844_846delAAC (Figure 3(a)). This nonframeshift mutation results, at the protein level, in a deletion of the asparagine amino acid at position 282 (p.Asn282del). Segregation analysis showed that the two parents were heterozygous for this mutation.

The p.N282del mutation had not been reported previously in LAD1 disease; it is not present in gnomAD exome, in genome databases, and in 96 control individuals from Morocco, belonging to the same North African population as the studied patient. Furthermore, it affects a highly conserved codon across multiple species (Figure 3(b)).

### 3.4. Bioinformatics Analysis

Analysis of this ITGB2 novel mutation by the in silico PROVEAN software yielded a score of -12.65, showing that it is deleterious. The MutationTaster yielded a score of 0.99 classifying it as disease-causing (Table 1).

**Figure 4:**
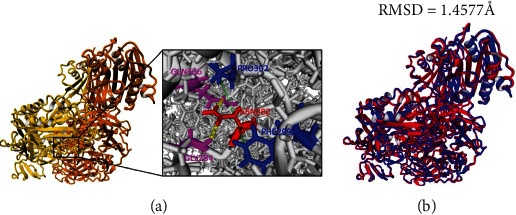
Bioinformatics analysis of the structural impact of the in-frame deletion on the integrin beta-2 and integrin alpha-X (CD11c) protein complexes. (a) Interaction analysis between Asn282 and neighboring amino acids. Integrin beta-2 protein is highlighted in yellow, and integrin alpha-X is highlighted in orange. Residues involved in hydrogen bonds are shown in magenta, and residues involved in hydrophobic interactions are shown in blue. Green lines between amino acids represent hydrophobic interactions, and yellow-dotted lines represent hydrogen bonds. (b) Superimposition of wild-type (blue) and mutated (red) complexes.

To further investigate the impact of this mutation on the protein structure and function, we performed a molecular modeling analysis of the wild-type integrin beta-2 in complex with integrin alpha-X (CD11c). The results showed that the asparagine in position 282 might play an important structural role. It interacts with two residues Gln306 and Gly284 through hydrogen bonds and is implicated in two hydrophobic interactions with Phe299 and Pro302 (Figure 4(a)). Therefore, based on this in silico analysis, the loss of these interactions due to Asn282 deletion may affect the structure of integrin beta-2 and subsequently its interaction with integrin alpha-X.

To evaluate the potential structural change upon Asn282 deletion, a superimposition was performed between native and mutation protein complexes. The distance between both complexes was 1.4577 Å (Figure 4(b)). This change in 3D structure may affect the protein complex function.

## 4. Discussion

Here, we report a Mauritanian family with a 5-month-old boy who presented with recurrent infections. Clinical and paraclinical investigations and family history suggested LAD1 disease, which was confirmed by flow cytometry showing reduced expression of CD18/CD11 glycoprotein. The hyperleukocytosis shown in the blood count of this patient is generally observed within severe LAD1 patients. In fact, although the alternative mechanisms for leukocyte emigration, including movement by chimneying, the dominant role of *β*2 integrins in adhesion and transmigration of human leukocytes is manifested by the dramatically impaired accumulation of myeloid leukocytes at extravascular sites in this affection [15, 16]. The successive occurrence of several episodes of infections observed within this patient is very common since the most frequently described infections in LAD1 cases with CD18 less than 2% are respiratory tract (including pneumonia), sepsis, and otitis media [4].

Sanger sequencing of the responsible gene *ITGB2* identified a novel homozygous in-frame deletion in exon 7, c.844_846delAAC (p.Asn282del) in the patient, for which the healthy parents were heterozygous. This mutation deletes a highly conserved codon across multiple species, which was absent from all genome and exome databases and also from control individuals from North Africa.

This novel mutation was predicted as disease damaging by the pathogenicity prediction tools used. Furthermore, molecular modeling analysis showed that the deletion of Asn282 may affect the structure of integrin beta-2 and subsequently its interaction with integrin alpha-X. Moreover, the Asn282del is localized in the same codon where two already pathogenic variants p.Asn282Lys and Asn282ThrfsX41 were described [17].

The p.Asn282del is located in a highly conserved protein domain from 124 to 364 amino acids called Von Willebrand Factor type A encoded by exons 5 to 9, where most of the known pathogenic mutations are located. This domain contains contact sites between *α* and *β* subunits and has an essential role in synthesizing subunit precursors [3, 17].

*ITGB2* mutations affect the expression level of *β*2 integrins on the leucocyte membrane ranging from reduced to complete absence without adhesive capacity [3, 10, 18]. This expression variability was associated with disease severity, and patients with less than 1% expression were clinically severe, whereas patients with 3 to 10% expression showed a moderate phenotype [4, 17]. Our patient showed CD11/CD18 expression of less than 2% and a very severe form of LAD1. The prognosis for long-term survival of such patients is poor and requires rapid therapeutic management.

Therefore, CD11/CD18 expression was considered as a marker for clinical diagnosis and disease severity of LAD-1 [19–21] and should be used as a first-line diagnostic approach in patients suspected to have LAD disease. Furthermore, the results of these initial immunophenotyping and functional assay tests could direct the geneticist to target quickly the responsible gene and give molecular confirmation to the clinicians to start therapeutic management.

The treatment of choice for LAD1 disease is immune reconstitution with hematopoietic stem cell transplantation [22]. A HLA typing of patient III.3 and his older brother III.1 was carried out to provide an allograft. However, it showed an absence of histocompatibility (5/10), and therefore, there was no current possibility of genoidentical allograft. For now, a long-term prophylactic antibiotic therapy has been established.

## 5. Conclusions

In conclusion, we identified a novel nucleotide variant (NM_ 0000211.5: c.844_846delAAC, p.Asn282del) in *ITGB2* in a proband diagnosed with severe LAD1 disease. Our study expands the spectrum of *ITGB2* mutations and may contribute to genetic diagnosis and counseling of families with LAD1 in North Africa.

## Figures and Tables

**Table 1:** In silico mutation analysis by PROVEAN and MutationTaster tools of the Asn282del mutation.

Gene	Reference sequence	Location	DNA Change	Amino acid variation	PROVEAN^∗^	MutationTaster^∗^
*ITGB2*	NM_000211.5	Exon 7	c.844_846delAAC	p.Asn282del	Deleterious (-12.65)	Disease causing (0.99)

^∗^Prediction (score).

## Data Availability

The data used to support the findings of this study are all included within the article.
